# Aging and tumors: a dynamic interaction

**DOI:** 10.1007/s12672-025-01808-9

**Published:** 2025-01-21

**Authors:** Yudi Zhang, Siqiang Zhu, Zhaodong Liu, Yonghao Hu, Yongjian Zhang, Liang Shang, Leping Li

**Affiliations:** 1 Present Address: Department of Gastrointestinal Surgery, Shandong Provincial Hospital Affiliated to Shandong First Medical University, Jinan, 250021 Shandong China; 2 Department of Gastrointestinal Surgery, Medical Science and Technology Innovation Center, Shandong First Medical University , Jinan, 250021 Shandong China; 3 Department of Gastrointestinal Surgery, Shandong Provincial Hospital, Shandong University, Jinan, China; 4 Department of Gastrointestinal Surgery, Key Laboratory of Engineering of Shandong Province, Jinan, 250021 Shandong China

**Keywords:** Aging, Tumor, Cellular senescence, Telomere length, Anti-aging treatment

## Abstract

Aging is an inevitable physiological process in organisms, and the development of tumors is closely associated with cellular senescence. This article initially examines the role of cellular senescence in tumorigenesis, emphasizing the correlation between telomere length—a marker of cellular senescence—and tumor risk. Concurrently, the study explores the expression levels of senescence-associated markers, such as p16, p53, and mTOR, in the context of tumor development. Additionally, the study investigates the impact of tumors on cellular and organismal senescence, including the effects on immune system function and metabolic processes. Ultimately, the discussion explores the potential application of anti-aging strategies in tumor therapy and considers the possibility of utilizing senescence mechanisms as a novel therapeutic approach for tumors. This research provides novel insights into the complex interplay between senescence and tumor development, suggesting potential strategies for future preventative measures and therapeutic interventions.

## Introduction

Senescence is widely recognized as a reflection of the diminishing capacity of an organism to adapt to environmental conditions, a process that encompasses both physiological and pathological elements. At the cellular level, senescence is characterized by a persistent cell cycle arrest, culminating in a permanent non-proliferative state instead of cell death [1]. Cellular senescence can be classified into various types based on its underlying mechanisms, including replicative senescence [2], senescence induced by Deoxyribonucleic Acid (DNA) damage response [3], oncogene-induced senescence [4], and oxidative stress-induced senescence [5] and so on. These classifications facilitate a comprehensive understanding of the mechanisms underlying cellular aging and provide a foundation for targeted therapeutic strategies. Spanish researchers have identified the hallmarks of cellular senescence and have categorized them into three distinct groups, comprising a total of twelve hallmarks. These include the basic category, which encompasses negative effects; the antagonistic category, exhibiting beneficial effects at low levels and detrimental effects at high levels; and the integrative category, affecting tissue homeostasis and functionality [6]. This taxonomy provides a comprehensive framework for understanding the complex mechanisms underlying cellular aging and their implications for tissue health and disease progression. Cellular senescence serves as a protective biological mechanism [7, 8], When normal cells encounter environmental stressors, including DNA damage and oxidative stress, they initiate the senescence program and transition to a senescent state to prevent further proliferation of damaged cells [9]. This response is crucial for maintaining tissue integrity and avoiding the propagation of potentially harmful mutations.

Tumors can be considered as new organisms formed when the body, under the action of unfavorable factors, loses the normal regulation of the growth of the cells of local tissues at the genetic level, leading to their clonal abnormal proliferation. In cellular biology, genetic damage can occur in individual cells following exposure to biological or physicochemical stressors. This damage leads to the activation of oncogenes and the inactivation of tumor suppressor genes, thereby disrupting the regulation of the cell cycle. Such alterations enable the mutated cell to evade growth regulation and circumvent immune detection, culminating in the formation of neoplastic tissue [10]. Currently, it is widely accepted that the genes implicated in tumorigenesis include proto-oncogenes, oncogenes, and tumor suppressor genes. These genes maintain a balanced and stable state within normal organisms and collectively uphold cellular homeostasis by regulating the cell cycle. Proto-oncogenes constitute a class of genes responsible for encoding proteins that regulate cell growth and division. When expressed at low levels, these genes modulate cell proliferation and division, sustaining cells within a defined size and morphological parameters, and orchestrating cell division at precise temporal intervals. Upon heightened expression levels, such as post-injury, they can transform into oncogenes, leading to the potential for cellular transformation into a malignant state [11]. All life activities of the organism are in a state of dynamic equilibrium, and cell growth, proliferation and differentiation also require cell cycle homeostasis. In addition to proto-oncogenes that stimulate the cell cycle, there is a class of genes capable of inhibiting unwarranted cell growth and proliferation, thereby suppressing tumor formation. These genes are referred to as tumor suppressor genes [12]. Tumor formation essentially represents a process of cellular adaptation to novel environmental conditions. However, these conditions are deleterious, inducing a state of uncontrolled cell proliferation, inhibiting cellular differentiation and cell death inhibition.

**Fig. 1:**
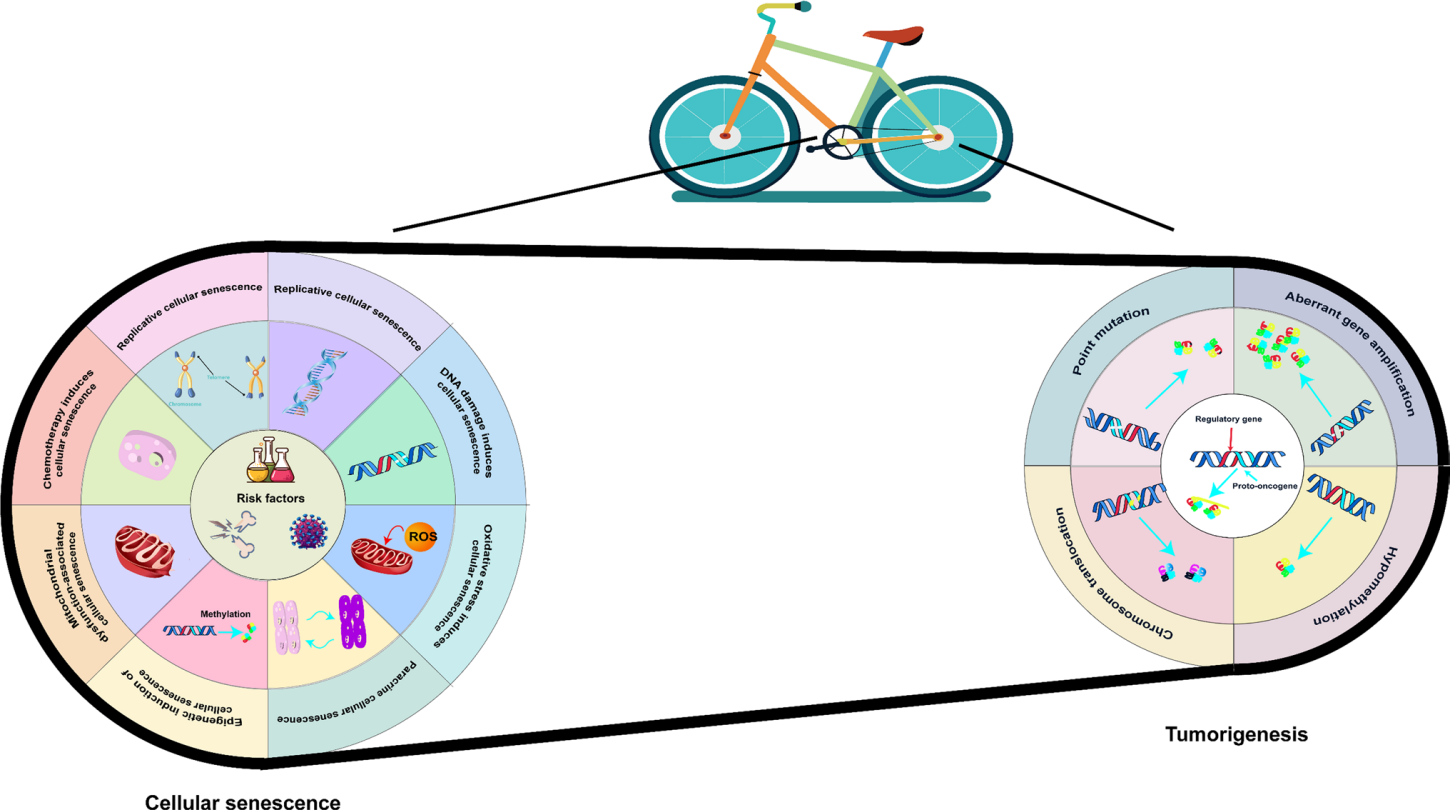
Potential mechanisms of association between Cellular senescence and Tumorigenesis. The left section describes various factors that induce cellular senescence, such as oxidative stress (Ros), DNA damage, epigenetic regulation, and mitochondrial dysfunction. The right part highlights the processes of gene mutation, aberrant gene amplification, and chromosomal rearrangements in tumorigenesis. The overall expression of cellular senescence and tumorigenesis under the influence of multiple factors may form a complex biological network relationship, the two have the potential to transform each other

In the life cycle of normal cells, growth, senescence, and death are the inevitable outcomes. The expression of proto-oncogenes and tumor suppressor genes collaborates to regulate cell growth. If a cell endures physical or chemical damage, it may also be subject to regulation by senescence-associated or oncogenic genes. Upon damage, the organism employs mechanisms to prevent the propagation of damage. Consequently, senescence and tumorigenesis are integral processes in the life cycle, exhibiting a dynamic interplay rather than being isolated in time and space. Understanding these complex dynamics is essential for developing therapeutic strategies aimed at modulating cellular aging and combating neoplastic transformations. We may be able to find interactions between aging and tumors in their characteristics (Fig. 1).

## Aging and tumor occurrence

### Relationship between aging and tumor

Tumor formation is a highly serendipitous process. The ways in which proto-oncogenes become oncogenes after damage include: mutations, gene amplification and chromosomal rearrangements [13]. Conversion of proto-oncogenes to oncogenes alone is not sufficient to produce tumors, and continued activation of oncogenes is another important cause of tumor production. The persistent activation of oncogenes necessitates interactions with promoters and enhancers, chromosomal translocations and rearrangements, and amplification of the gene sequence. In thermodynamic terms, the probability of a base mismatch is one in a hundred; however, due to the selective and corrective actions of enzymes, the intricate architecture of replication forks, and the presence of DNA repair mechanisms, the chance of a base mismatch becomes 10^–10^ [14]. According to modern analyses of human genome-wide data, the mutation rate of bases in normal DNA is only one to three parts per billion [15]. In summary, the development of tumor is the result of a confluence of various factors.

Senescence is characterized by an injury-induced stress response that triggers the activation of a diverse array of cytokines and chemokines, along with the secretion of inflammatory and growth-promoting factors by senescent cells following cell cycle arrest. This constellation of secreted factors is collectively referred to as the senescence-associated secretory phenotype (SASP) [16]. Multiple theories have been proposed to explain the mechanisms underlying aging, including growth metabolic signaling pathways, oxidative stress, and chronic inflammation. Single theory can’t account for all manifestations of aging, aging results from a complex interplay of various mechanisms [17]. We may be able to find an interaction between aging and tumors in their characteristics.

Podolskiy et al. identified a pattern of senescence-associated mutations through the analysis of the human tumor genome, and discovered that the number of such mutations approximately doubles every 8 years, consistent with the approximate 15-year latency period observed in tumorigenesis. This suggests that aging may be an important risk factor for tumourigenesis [18]. In addition, aging and tumors share similar features, including genomic instability, epigenetic changes, chronic inflammation and ecological dysregulation [6]. Damage to DNA hinders the accuracy, control and conservation of genetic information. Damaged DNA can counteract genetic erosion by the action of the repair system, however, the repair system can also be affected in the process of DNA damage, and become a factor in genetic instability [19].

Hutchinson-Gilford Progeria Syndrome (HGPS) is a rare and fatal genetic disorder characterized by developmental delays and progressive senile degenerative changes beginning in infancy. It is caused by mutations in the *LMNA* gene, leading to the accumulation of an abnormal prelamin A protein known as progerin, which disrupts nuclear structure and function. In HGPS, which is caused by a genetic mutation, a point mutation in the ‘Lamin A’ gene incorrectly codes for thymine (T) wherever there is a cytosine (C), resulting in Lamin A that is 50 amino acids short [20]. At the same time, two genes, Cockayne syndrome A (CSA) and Cockayne syndrome B (CSB), which are related to DNA repair and replication and translation functions, were discovered. Defects in the CSA and CSB genes prevent the body of the patient from carrying out normal DNA repair work, and the body loses its ability to replicate cells and make proteins [20]. In the Cancer Genome Atlas (TCGA) published by the journal ***Cell***, defects in DNA damage repair have been identified as one of the causes of tumor development. Breast cancer susceptibility genes (BRCA) are significant tumor suppressor genes and markers of cancer risk in the human body. The functional proteins regulate cell growth and prevent the proliferation of abnormal cells with potential carcinogenicity. These proteins are involved in repairing double-strand DNA breaks via homologous recombination pathways, which is crucial for maintaining genomic integrity [21]. However, mutations in BRCA genes result in dysfunction of the BRCA proteins and impaired DNA damage response (DDR) repair, thereby significantly increasing genomic instability and predisposing individuals to various cancers, including those of the breast, ovary, and prostate [22].

Epigenetic alterations involve chemical modifications that alter DNA and chromosomal proteins, thereby affecting gene expression. Common types of these modifications include DNA methylation, histone tail modification, chromatin remodeling, and non-coding Ribonucleic Acid (RNA) [23]. Organismal aging is characterized by a reduction in DNA methylation levels and is also associated with hypermethylation of certain oncogenes [24]. Notably, human cancers exhibit extensive DNA methylation within their epigenetic landscapes [25]. Chromatin remodeling factors such as Heterochromatin protein 1α (HP1α) [26]、Switching Defects (SWI) /Sucrose Non-Fermentable (SNF) family [27]and Polycomb group (PcG) proteins, may mitigate the progression of aging and cancer. Dysfunction of these elements can lead to disruptions in chromatin structure, resulting in the loss and redistribution of heterochromatin levels overall. The histone Histone 3 lysine 27 trimethylation (H3K27me3) demethylase ubiquitously transcribed tetratricopeptide repeat, X chromosome (UTX) has been identified as a significant regulator of senescence in Hidradenitis elegans nematodes. Both heterozygous mutants and wild-type RNA knockdown of this gene significantly extended nematode lifespan and enhanced its resilience substantially. Genetic analysis revealed that its function is contingent upon the insulin-like signaling pathway. This mechanism of action, which involves re-establishing histone modification patterns, underscores the pivotal role of cellular reprogramming in counteracting the aging process [28]. H3K27me3 methyltransferase is upregulated in a number of cancers, including prostate cancer [29], lymphoma [30]and ovaries [31].Notably, activating point mutations in EZH2 have been linked to B-cell lymphomas, supporting the notion that Enhancer of Zeste Homolog 2 (EZH2) possesses oncogenic properties [32]. In line with these findings, somatic inactivating point mutations within the Histone 3 lysine 27 (H3K27) demethylase UTX gene have been discovered across multiple cancer types.

At the molecular level, cellular senescence is characterized by the SASP, which induces chronic inflammation and impels neighboring cells toward senescence [33]. At the cellular level, immune cells act as scavengers that eliminate senescent cells. With aging of the immune system, its capacity to clear senescent cells and pro-inflammatory factors diminishes, resulting in increased inflammation. The resultant chronic inflammation exacerbates senescence, perpetuating a vicious cycle. Cytokines produced during chronic inflammation can induce gene mutations, modulate the expression and transformation of oncogenes, inhibit apoptosis, promote neovascularization, and disrupt inflammatory signaling pathways. Chronic inflammation facilitates the creation of an immunosuppressive tumor microenvironment (TME) by recruiting various immune-suppressive cells (e.g., M2-TAMs, MDSCs, Tregs), thereby contributing to tumor development and progression [34].

DDR acts as a trigger for aging and tumorigenesis, compromising genomic stability and consequently inducing epigenetic alterations. This phenomenon, in conjunction with the detrimental effects of chronic inflammation and ecological imbalance, perpetuates a cycle of aging and tumorigenesis. The impact of aging on tumorigenesis is complex and dualistic. As a cell approaches the Hayflick limit following damage, the initiation of the senescence program can prevent the propagation of damaged genes, thus serving as a barrier to tumorigenesis and progression. Paradoxically, more studies have demonstrated that malignant and non-malignant cells with persistent senescence can acquire tumorigenic properties [35]. Once metastasis occurs, the prognosis and survival rates of cancer patients are significantly diminished. Studies have demonstrated that senescent cells exhibit reduced resistance to the invasive behavior of cancer cells, and that cellular senescence promotes the metastasis of cancer tissues, resulting in an unfavorable prognosis for cancer patients [35].

### Telomere length and tumor development

In eukaryotic cells, the ends of linear chromosomes do not contain genetic information, but rather a short, repetitive, non-transcribed sequence and a number of binding proteins that form a special structure that safeguards chromosome ends from fusion and degeneration. Chromosome localization, replication, protection, and regulation of cell growth and lifespan are critically important functions. However, telomeres do not remain intact with each chromatin replication; they undergo gradual shortening following each cell division. When telomeres are entirely eroded, the cell begins to be damaged and enters the death phase. Thus, telomere miniaturization is a signal of cellular aging or the ability of cells to divide is dependent on a certain length of telomeres [36].

In the majority of cancer cells, telomerase activity is induced, appending telomeric sequences to telomeres, thereby preserving telomere length stability and sustaining the cell's capacity for continued division. This phenomenon is referred to as the Telomere Maintenance Mechanism (TMM), which is crucial for the uncontrolled proliferation characteristic of cancer cells and contributes to their resistance to cellular senescence [37]. Research indicates that a pivotal event in tumorigenesis is the upregulation or reactivation of telomerase [38]. The majority of tumor biopsies exhibit telomerase positivity, which can be as high as 95% or more in lung cancer [39]. Telomerase can induce uncontrolled cell proliferation through mechanisms that include senescence evasion and cellular immortalization. However, telomerase is not a direct instigator of cellular carcinogenesis; its activation typically follows the initiation of carcinogenesis. Upon the genesis of the initial tumor cell, telomerase activation endows the cell with an exceptional capacity for growth, proliferation, and indefinite division [40]. Most tumors maintain telomeres by activating telomerase, whereas others, such as glioblastoma multiforme, extend telomeres through homologous recombination, an alternative mechanism for telomere elongation [41].

The widely accepted notion is that shorter telomeres elevate the risk of cancer by contributing to chromosomal instability. Nonetheless, a long-term study of individuals with short telomere syndrome, which results in premature aging, along with murine experiments, involved sequencing the complete genomes of eight patients with squamous cell carcinoma across various organs. However, chromosomal instability, characterized by translocations or fragmentation, was not observed. In contrast, the chromosomes of the squamous cell carcinoma patients in this study seemed to exhibit greater stability compared to those without the short telomere syndrome. Subsequently, the team analyzed the immune systems of these squamous cell carcinoma patients, discovering a general depletion of T-cells. Ultimately, they confirmed their hypothesis in a mouse model, demonstrating that T-cell immunodeficiency, rather than chromosomal instability attributable to telomere shortening, increases cancer susceptibility [42].

Sustaining cell proliferation necessitates the preservation of telomeres at a critical length. The capacity for indefinite division is a hallmark of tumor cells, which rely on an efficient telomere maintenance mechanism to attain immortality. A subset of cancers is characterized by the activation of telomerase, while a minority utilizes the alternative lengthening of telomeres (ALT) pathway. An emerging perspective suggests that a portion of cancers may be independent of telomere involvement.

### Senescence-related markers in tumors

To objectively quantify aging, numerous biomarkers associated with the aging process have been identified, and corresponding assays have been established. Genetically, the extent of DDR can be ascertained by assessing the expression levels of phosphorylated histone H2AX (p-γH2AX), Ataxia Telangiectasia and Rad3-Related Protein(ATR) [43]. The cellular state of cell cycle arrest is confirmed by evaluating the expression levels of p15, p16, p21, p53, or hypophosphorylated pRb protein [44]. The expression of SASP components, such as interleukins, chemokines, and non-protein molecules, can also be used to assess cellular senescence [45, 46]. Cellularly, both cellular morphology and the organism's overall metabolic state can be examined. Given that genetic damage is an initiating factor in both senescence and tumorigenesis, we may be able to explore the role of aging in tumors at the genetic level.

As a pivotal component of the DDR pathway, the p53 tumor suppressor protein also serves as a crucial regulator of the cell cycle. The accumulation of p53 promotes the activation of cell cycle protein-dependent kinase inhibitor (CDKI), culminating in cell cycle arrest [47]. The senescence marker p21 is a CDKI downstream of p53. P21 serves as a pivotal cell cycle regulator, impeding the cell's progression from the G1 to S phase by interacting with Cyclin-Dependent Kinase 2 (CDK2) [48]. p53 is a tumor suppressor gene that primarily maintains cellular and tissue stability through mechanisms such as DNA damage response, cell cycle regulation, apoptosis, and cellular senescence [49]. By activating cellular senescence pathways (e.g., via p21 and PML proteins), p53 limits cellular proliferation, leading to an irreversible state of growth arrest. Under conditions of intense or sustained DNA damage, excessive activation of p53 may result in stem cell depletion and a decline in tissue regenerative capacity, thereby accelerating overall aging processes. Additionally, the regulation of p53 activity plays a critical role in balancing aging and healthspan. Low p53 activity may lead to genomic instability and increased risk of tumorigenesis, whereas high p53 activity may induce premature cellular senescence and compromise stem cell function [50]. Mutations in the p53 gene represent significant targets for cancer development, progression, treatment, and prognosis. However, a study examining the role of p53 in the context of cancer development by Baslan et al. determined that p53 deletion alone is insufficient for cancer induction; rather, P53-deficient cells must acquire additional genetic changes in an orderly fashion, uncontrollably proliferate and ultimately transition to cancer [51].

The expression of p16, a widely recognized and reliable biomarker of senescence, is believed to contribute significantly to the process of cellular senescence [52]. p16, a member of the inhibitor of CDK4 (INK4) family, plays a crucial role in cell cycle regulation. It interacts with cyclin-dependent kinases 4 and 6 (CDK4/6), thereby inhibiting the cell cycle in the G1 phase. Under normal conditions, the p16 gene functions to inhibit tumor growth by regulating cell cycle progression and responding to cellular stress signals. Studies have identified epigenetic mutations in the p16 gene as potential new therapeutic targets for rectal cancer treatment [53]. In this instance, p16 would not be silenced by a mutation in the gene's own gene, but rather by a different process of epigenetic mutation. A study introduces an adenomatous polyposis coli (APC) gene mutation into an animal tumor model, which initiates the growth of benign tumors and epigenetic modifications, such as methylation of the p16 gene. Analysis of tumor development and progression demonstrated that mice with both the APC mutation and p16 epigenetic alteration exhibited significantly reduced survival times and enhanced tumor growth compared with mice harboring the APC mutation alone [53]. Mutations in the p16 gene substantially contribute to tumor development and can lead to a reduction in the organism's lifespan.

Typically, pRB restricts cell proliferation and functions as a tumor suppressor gene during the cell cycle, playing a role in regulating the cell cycle in response to cellular damage. Cell cycle inhibition by various CDKIs, including p21 and p16, results in the hyperactivation of Rb, ultimately culminating in cell cycle arrest and senescence [54]. Loss of pRB function is common across all cancer types and is particularly pronounced in pediatric malignant retinoblastoma, where 98% of patients exhibit a loss of pRB function [55].

Senescence-associated cell cycle regulatory genes are highly relevant to cancer development. On one hand, the silencing of oncogenes, such as p16 and p53, significantly influences cancer development and progression. On the other hand, the majority of cancer-associated DNA methylation alterations predominantly impact introns or intergenic regions, with methylation of the p16 gene potentially exacerbating the reduced survival times of cancer patients.

## Impact of tumors on aging

### Tumor effects on cellular senescence

The various normal cell groups within an organism exhibit a state of specialized division of labor and mutual harmony. However, upon emergence, tumor cells compete with normal cells for scarce resources and space within the tissue [56], thereby acting as resource predators. Zhou, Jun et al. analyzed gene expression profiles of intestinal tumors and corresponding normal tissues, discovering that tumor cells exert mechanical competitive pressure that displaces surrounding normal cells. This displacement activates the immune-responsive NF-κB pathway, leading to the segregation and apoptosis of adjacent normal cells and consequently fostering tumor growth.

In the 'N-Akt' mouse liver cancer tumor model, elevated levels of Yes-associated protein (YAP) and transcriptional co-activator with PDZ-binding motif (TAZ) were observed within the N-Akt tumor cells, with substantial YAP expression also noted in the hepatocytes adjacent to the N-Akt tumor cells. Conversely, YAP expression was barely detectable in hepatocytes from normal livers. In conclusion, YAP and TAZ are indeed activated in normal cells surrounding the tumors. This activation leads to a downregulation of YAP and TAZ in normal hepatocytes adjacent to the tumors, resulting in an increase in tumor volume in mice [57]. Stefano Piccolo and colleagues discovered that YAP/TAZ activity in stromal cells declines during physiological aging, with the loss of YAP/TAZ activity accelerating the aging process. In contrast, sustaining YAP function can restore cellular vitality and inhibit the onset of aging-associated characteristics [58]. This finding suggests that YAP and TAZ, despite being considered as having pro-oncogenic effects, may also exert anti-oncogenic effects. Following tumorigenesis, the tumor can influence surrounding cells, altering their gene expression and potentially inducing resistance to tumor cell proliferation [59].

Tumors accelerate cellular senescence through various mechanisms [60], including inducing mitochondrial dysfunction. The tricarboxylic acid (TCA) cycle, also known as the citric acid or Krebs cycle, is a core metabolic pathway responsible for energy production and the generation of biosynthetic precursors. Due to their rapid growth and high metabolic activity, tumor cells undergo metabolic reprogramming to meet their abnormally high demands for energy and nutrients. The metabolic adaptability of tumor cells manifests in several ways: they sustain survival under hypoxic conditions through aerobic fermentation and lactate production, regulate metabolic processes by altering the activity of key enzymes in the TCA cycle, and support abnormal metabolism through mitochondrial reprogramming, thereby accelerating tissue aging [61]. Mitochondrial mutations, which play critical roles in certain cancers such as prostate and hepatocellular carcinoma, disrupt energy metabolism and cell proliferation. Mutations in succinate dehydrogenase (SDH), a key enzyme complex in the TCA cycle, result in the production of reactive oxygen species (ROS) and the accumulation of succinate, which disrupts TCA cycle function and leads to metabolic dysfunction [62]. Additionally, SDH mutations inhibit the activity of hypoxia-inducible factor (HIF) prolyl hydroxylase, preventing the degradation of HIF proteins and leading to the activation of downstream signaling pathways. The HIF pathway regulates critical processes such as angiogenesis, metabolism, cell cycle progression, and anti-apoptosis, thereby promoting tumor initiation and progression [63].

Tumors also suppress immune responses by exhausting T cell function through immune evasion mechanisms, resulting in immunosenescence-like states [64]. Immunosuppressive factors in the tumor microenvironment, such as TGF-β, further disrupt immune homeostasis and cause systemic immune dysregulation. Moreover, tumor cells release extracellular vesicles carrying oncogenes and inflammatory signals, which can remotely induce senescence in normal cells [65]. These combined effects contribute to both cellular and systemic aging, as well as tumor progression.

### The effect of tumors on the overall aging of the organism

Tumors are not merely localized pathological entities; they profoundly impact systemic aging through a variety of mechanisms. First, inflammaging, a chronic low-grade inflammatory state, plays a pivotal role. Within the tumor microenvironment, tumor cells and associated stromal cells, such as macrophages and fibroblasts, secrete pro-inflammatory cytokines (e.g., IL-6, TNF-α, IL-1β). These cytokines not only promote tumor progression but also disseminate systemically, leading to chronic inflammation, which damages tissues, cells, and organs, accelerating systemic aging [66]. Second, tumor-secreted pro-inflammatory and catabolic factors drive the breakdown of muscle and adipose tissues, causing severe nutritional imbalance and systemic energy depletion, a condition known as cachexia [67]. Additionally, chronic stress and pain induced by tumors can activate the hypothalamic–pituitary–adrenal (HPA) axis, resulting in the overproduction of stress hormones, such as glucocorticoids, which further contribute to immunosuppression, osteoporosis, and other aging phenotypes [68]. Furthermore, hormone-dependent cancers, such as breast and prostate cancer, may disrupt hormonal balance, leading to more extensive systemic aging effects.

## Anti-aging and anti-tumor therapy

### Anti-aging strategies in anti-tumor therapy

Given the biphasic role of senescence in tumorigenesis, it can be inferred that strategies aimed at either inducing senescence in tumor cells or preventing it in normal cells, or a combination thereof at precise targets, hold promise for developing novel anticancer therapies. Notably, in the early stages of treating tumors that are refractory to conventional removal, medical professionals have historically employed cancer therapies designed to induce senescence or apoptosis in tumor cells, namely chemotherapy and radiotherapy. Chemotherapy and radiotherapy share the mechanism of inducing genetic instability in tumor cells at high doses, resulting in cellular apoptosis. However, these treatments are not without limitations. On one hand, tumor cells that evade senescence may exhibit increased aggressiveness, stem-like characteristics, and drug resistance, potentially leading to metastasis and tumor recurrence [69]. On the other hand, while high-dose chemotherapy or radiotherapy impedes tumor cell proliferation, it can also induce cell death in normal tissues throughout the body or in the vicinity. This highlights the need for therapeutic strategies that are not only effective at targeting tumor cells but also minimize damage to healthy tissue [70]. The possibility of developing a therapeutic agent that selectively targets and eliminates tumor cells without adversely affecting other cells is affirmative, and such targeted therapeutics already exist. The identification of oncogenes has been pivotal for the development of these targeted therapeutics. These targeted therapeutics are designed to selectively bind to the known oncogenic loci upon entering the body, thereby disrupting cancer cell proliferation, development, and metastasis. This selective action leads to the apoptosis of tumor cells while sparing the surrounding normal tissue, culminating in the targeted eradication of the tumor [71]. However, targeted therapeutics do not exhibit efficacy in all patients [72]. In individuals harboring tumors with driver-sensitive mutations, targeted therapies elicit remarkable responses. Conversely, in patients with tumors lacking such mutations, the therapeutic outcomes are modest.

**Fig. 2:**
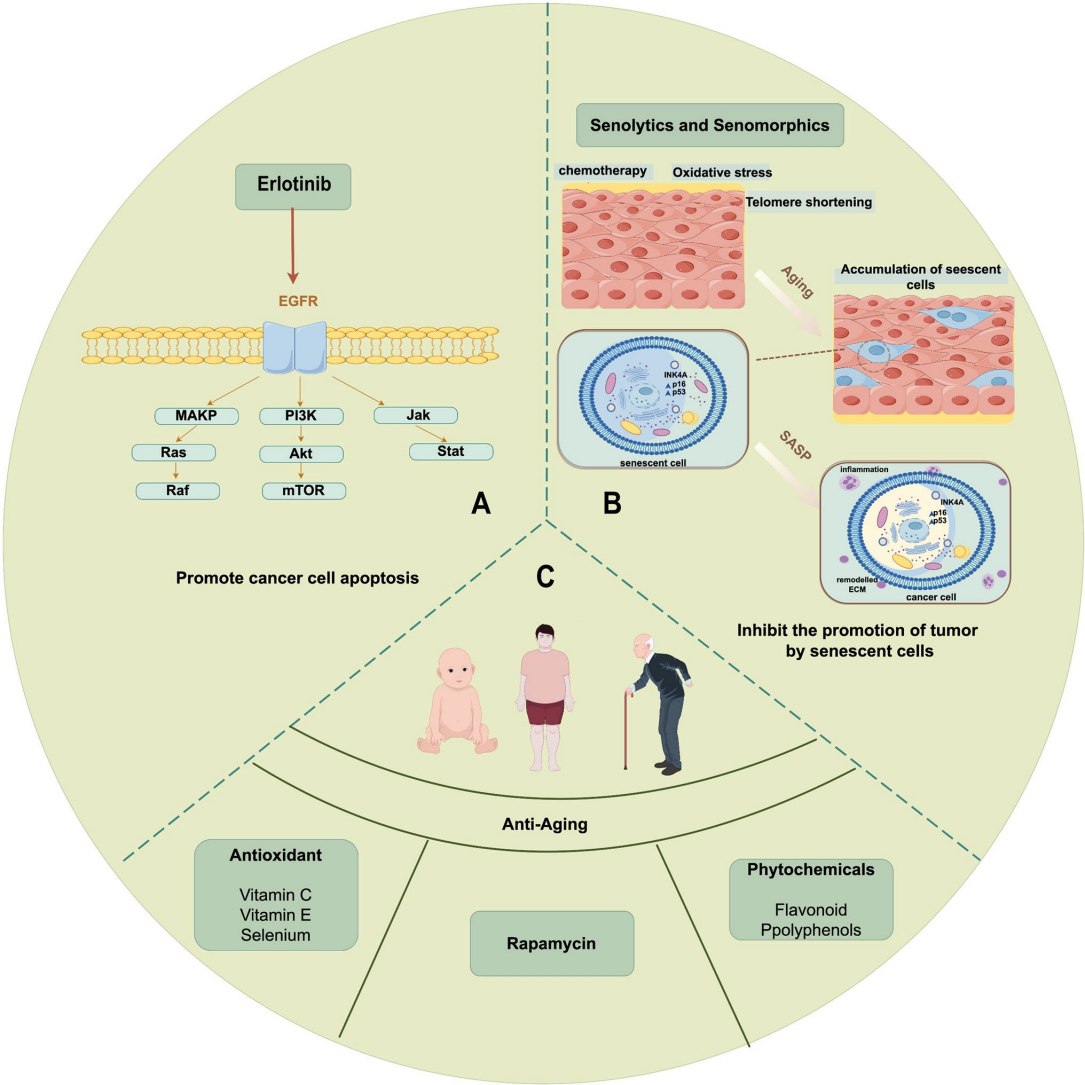
Strategies for treating tumors using the relationship between cellular senescence and tumors. **A** Anti-tumor drugs represented by Erlotinib that affect the cellular aging pathway. **B** SASP produced by senescent cells is used to exert anti-tumor effects. **C** Anti-aging strategies (antioxidant supplementation, rapamycin, and phytotherapy) affect tumors

The development of antitumor drugs leveraging aging-related mechanisms represents a novel frontier in oncology (Fig. 2). Initially, recognizing the shared meta-features of aging and tumorigenesis reveals analogous developmental pathways. Subsequently, aging, as a chronic process, diminishes the resilience of tumor cells to radiation or pharmacological agents, thereby facilitating their demise. Ultimately, antitumor drugs that exploit senescence pathways exhibit reduced systemic toxicity and increased therapeutic index. Senolytic therapies targeting senescent cells are increasingly recognized as a safer alternative to conventional radiotherapy and chemotherapy, offering new hope for patients with cancer [73].

Epidermal Growth Factor Receptor (EGFR), a receptor tyrosine kinase, activates signaling pathways that regulate cell proliferation and division upon stimulation [74]. EGFR inactivation can lead to the suppression of cell growth and proliferation. EGFR activation suppresses apoptosis, while its inactivation abrogates this protective effect, rendering cells more prone to undergo programmed cell death. Mutations within the EGFR gene are prevalent in lung cancer. Such mutations can result in the constitutive activation of EGFR, thereby promoting the uncontrolled proliferation of cancer cells [75]. Erlotinib suppresses the proliferation and metastasis of cancer cells by inhibiting EGFR activation and subsequently blocking downstream signaling pathways. This agent induces cellular senescence and apoptosis by disrupting the signaling pathways that regulate cell growth [76]. However, erlotinib also has adverse effects that affect digestive system [77] and cardiotoxicity [78].

Senescent cells undergo a state of cytostasis, characterized by an absence of cell division and evaded apoptosis, during which they SASPs that alter the extracellular milieu. SASPs facilitate the recruitment of immune cells to the TME, thereby potentiating the inhibition of tumor cell proliferation through their pro-inflammatory effects [76, 79]. Senomorphics therapies work by inhibiting signalling pathways that regulate the cellular aging-associated secretory phenotype SASP or individual SASP factors that promote aging in tumor cells. This strategy is aimed at mitigating the tumor-promoting effects and chemotherapeutic toxicity associated with cellular aging, while retaining the beneficial aspects of cellular aging on immunogenicity, particularly pertaining to tumor immunosurveillance during cancer cell senescence [80].

In the human body, the proportion of senescent cells can reach up to 15% of the total cellular population, and the targeted removal of these cells could confer significant benefits [81]. Senolytics can selectively target and eliminate senescent cells, or potentially reverse the aging process at the organismal level. Since their inception, senolytic therapies have demonstrated promising outcomes in preclinical animal models and initial clinical trials. Dasatinib exhibits a favorable therapeutic profile in the treatment of chronic myeloid leukemia (CML) through the inhibition of BCR-ABL kinases, including c-KIT, EPH receptor kinase, PDGFRβ, and SRC family kinases, among other oncogenic kinases [82, 83]. Combined treatment with Dasatinib and quercetin removes senescent cells in the short term, reduces senescent cell burden and decreases SASP secretion levels [84]. Procyanidin C1 has been evaluated across diverse human cellular models and in naturally senescent mice, and has exhibited the capacity to identify and eliminate senescent cells. Furthermore, it has been shown to suppress the secretion of SASP in cells undergoing senescence due to replicative exhaustion, oncogene-induced senescence, or exposure to senescent-inducing factors like radiation and chemotherapy [85].

Massive DDR activates ATR signaling pathways, resulting in the activation of p53 and p21. Vorinostat, a precise targeted HDAC inhibitor, is approved for the treatment of cutaneous T-cell lymphoma. It exerts antitumor effects through mechanisms such as regulating gene expression, inducing apoptosis, arresting the cell cycle, inhibiting angiogenesis, and reducing immune evasion [86]. By inhibiting HDAC activity, Vorinostat increases histone acetylation levels, leading to chromatin relaxation and alterations in gene transcription.In its antitumor capacity, Vorinostat activates tumor suppressor genes (e.g., p21), thereby promoting cell cycle arrest and apoptosis [87]. Additionally, it counteracts cellular aging by reshaping the epigenetic landscape and maintaining genomic stability. HDAC inhibitors can restore the normal expression of aging-related genes (e.g., suppressing the excessive expression of pro-inflammatory genes), thereby mitigating functional decline in aging cells. Vorinostat also regulates the expression of DNA damage repair genes (e.g., BRCA1 and ATM), reducing DNA damage accumulation and delaying the onset of cellular senescence [88].Vorinostat [89] or Decitabine induce CDKN2A expression [90], which activates p53-mediated and cell cycle protein-dependent kinase (CDK) inhibition-mediated senescence through transcripts encoding ARF and INK4A.

### Mechanisms of aging in tumors therapy

In addition to facilitating the induction of apoptosis in tumor cells as a therapeutic strategy, we can also concentrate on enhancing the genomic integrity of normal cells through interventions such as anti-aging pharmaceuticals or lifestyle modifications that mitigate genetic damage. Presently, research on anti-aging pharmaceuticals primarily aims to decelerate the aging process, extend lifespan, or ameliorate age-related conditions, rather than being explicitly tailored for oncological applications. Research into anti-aging pharmaceuticals may encompass mechanisms that are pertinent to oncology, thus overlapping with cancer research to a certain degree. This convergence suggests that therapies initially developed to address aging could potentially be repurposed or augmented to combat cancer, offering new avenues for investigation in oncology. For instance, rapamycin, an immunosuppressive agent initially utilized to prevent allograft rejection, has garnered interest in the field of oncology. Studies indicate that rapamycin exhibits inhibitory effects on specific cancer types and demonstrates antitumor potential in preclinical models [91]. Nonetheless, further extensive research and clinical trials are required to ascertain rapamycin's efficacy in cancer treatment.

Antioxidants are posited to possess cancer-preventive potential, due to their capacity to neutralize or mitigate free radicals within the body, consequently attenuating cellular damage induced by oxidative stress [92]. Free radicals, characterized by the presence of unpaired electrons, can induce cellular damage and contribute to the pathogenesis of various diseases, including cancer. However, the efficacy of antioxidants in tumor prevention remains inconclusive, with some studies suggesting potential protective effects against certain cancer types, including those attributed to vitamins C [93] and E [94], and selenium[95]. These antioxidants are posited to safeguard against DNA damage, thereby potentially diminishing cancer risk. Epidemiological analyses have indicated that an adequate intake of antioxidant-rich foods, including fruits and vegetables, correlates with a reduced cancer risk. Antioxidants neutralize or sequester free radicals, thereby stabilizing them and preserving cellular membrane integrity and normal cellular architecture and function [92, 96]. DNA damage inflicted by free radicals can precipitate genetic mutations and apoptosis. Antioxidants mitigate oxidative DNA damage, thereby preserving genomic integrity. Vitamin C is posited to suppress NF-κB activation and modulate inflammatory responses through the attenuation of oxidative stress. Glutathione, an endogenous antioxidant, preserves the stability of the p53 protein and facilitates its activation upon DNA damage [97]. Flavonoid compounds, exemplified by quercetin, are posited to possess antioxidant and anti-inflammatory properties and are hypothesized to exert their protective effects through the inhibition of the Mitogen-Activated Protein Kinase (MAPK) signaling pathway [98]. Antioxidants mitigate chronic inflammation induced by oxidative stress, consequently retarding the progression of cellular senescence. Although controversy exists within the literature, certain studies propose that elevated antioxidant intake during cancer therapy could correlate with adverse treatment efficacy, potentially due to their mitigating impact on the cytotoxic effects of radiotherapy or chemotherapy [99, 100]. A number of large-scale clinical trials have not shown a clear effect of antioxidants on cancer prevention. Furthermore, select investigations have indicated that high-dose beta-carotene supplementation may be correlated with an elevated risk of lung cancer [101]. The study in the United States found that supplementation of selenium and vitamin E may increase the risk of prostate cancer, depending on the individual's baseline selenium levels [102]. Although antioxidants have been widely used in clinical anti-aging, there is still insufficient evidence on the effects of antioxidant and trace element supplementation on tumors.

### The dual role of senescence in cancer

The role of senescence in cancer is highly complex and varies depending on the cancer type and tumor microenvironment. Significant controversies regarding the effects of senescence are particularly evident in the following cancer types: (a) Breast cancer: The SASP may promote cancer cell proliferation and invasion by secreting pro-inflammatory factors such as IL-6 and IL-8 [103]. Conversely, it has the potential to promote tumor cell metastasis [104]. While senescence induction in treatment may be beneficial in the short term, it could potentially lead to recurrence and therapy resistance in the long term. (b) Prostate cancer: Prostate cancer is androgen-dependent, and senescence may exert dual effects on cancer progression by altering hormonal balance and the tumor microenvironment [105]. Senescence-induced immunosuppressive microenvironments may undermine the effectiveness of immunotherapies. (c) Lung cancer: In non-small cell lung cancer (NSCLC), senescence may suppress cancer progression by limiting cell proliferation but could also exacerbate cancer cell survival and metastasis through pro-inflammatory signaling pathways [106]. Pemetrexed inhibits cell replication by disrupting key folate-dependent metabolic processes essential for DNA synthesis, thereby promoting premature cellular senescence to treat NSCLC [107]. A critical challenge lies in mitigating immune evasion and enhanced therapy resistance caused by senescence. (d) Pancreatic cancer: The microenvironment of pancreatic cancer is characterized by extensive fibrosis. SASP may exacerbate this condition, facilitating cancer cell adaptation to adverse conditions. However, it could also suppress tumor expansion through specific metabolic inhibitory signals [108]. The protective effects of senescence are often limited in highly malignant and rapidly progressing cancers. (e) Melanoma: SASP activation in melanoma may enhance the invasiveness of melanoma cells [46], particularly following targeted therapy [109]. Residual cells might re-emerge through SASP signaling. Whether senescence induction leads to drug resistance and relapse remains an unresolved question.

**Fig. 3:**
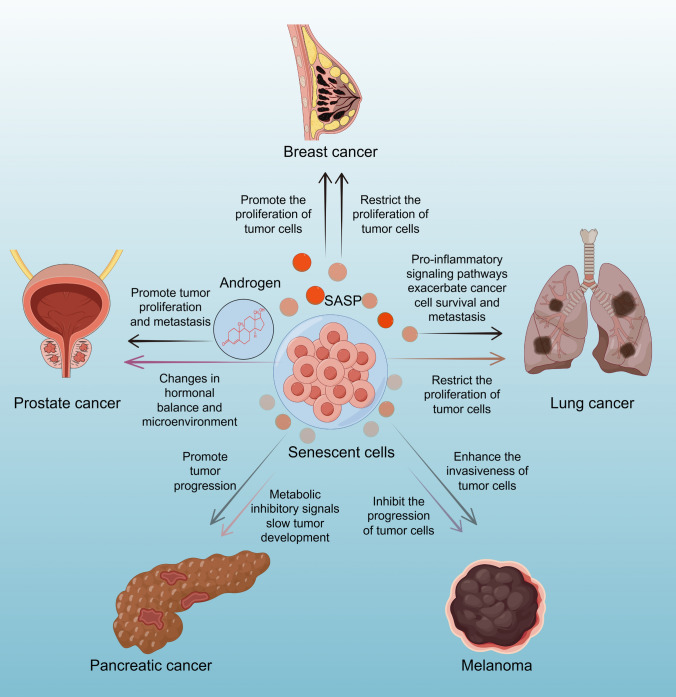
Double-sided effects of senescent cells in some tumors. SASP produced by senescent cells may promote the proliferation, invasion and immune escape of tumor cells, and may also trigger an inflammatory response to inhibit the development of tumor cells. Aging testes produce less androgen, which inhibits the development of prostate cancer

The dual role of senescence in suppressing or promoting cancer is determined by the cancer type and tumor microenvironment. Significant heterogeneity among individual patients renders the effects of senescence unpredictable. Furthermore, the short-term and long-term impacts of senescence induction are often divergent, posing potential therapeutic risks. Future research should focus on elucidating the molecular mechanisms underlying these controversies, particularly on how to modulate the positive and negative effects of SASP, to optimize senescence-targeted cancer therapies (Fig. 3).

## Conclusion

Cellular senescence, categorized as physiological and pathological, occurs continuously throughout the lifespan, fulfilling beneficial roles in embryonic development, tissue repair, and tumor suppression. Pathological cellular senescence exhibits similarities with the processes of tumorigenesis. Both phenomena originate from genomic instability triggered by environmental perturbations and share characteristics such as genomic instability, epigenetic alterations, chronic inflammation, and dysregulated cellular ecology, culminating in a predisposition to systemic debilitation. Aging and tumorigenesis can occur concurrently within the same spatial and temporal framework, exerting bidirectional influences on each other. Current research indicates that aging may exert paradoxical effects on tumorigenesis, as senescent cells can reduce tumor cell proliferation and impede tumor growth and metastasis. Conversely, senescent cells exhibit heightened vulnerability to environmental perturbations and display enhanced genomic instability, which can augment the risk of tumorigenesis and compromise the effectiveness of standard oncological therapies. Tumor cells exert effects on both their microenvironment and the host organism. On one hand, normal cells can impede tumor progression through interactions with tumor cells. On the other hand, tumor cells exploit normal cells to facilitate immune evasion and enhance metastatic potential. Therefore, how to use the link between aging and tumor cells to treat tumors still needs more in-depth research, that is, to use the beneficial effects of aging on tumor treatment to avoid the side effects caused by it.

The intricate interplay between aging and tumorigenesis compels a comprehensive investigation into therapeutic strategies; we must identify the bridge that contact this relationship, thereby potentially leveraging the aging process as a therapeutic intervention for tumors. It is evident that both aging and tumorigenesis are subject to genetic regulation and exhibit analogous effects. The direction of our research becomes clear. Existing strategies to inhibit tumor cells by intervening in their life cycle progression include kinase inhibitors that prevent tumor cells from entering the cell cycle, such as erlotinib and imatinib, as well as immune checkpoint inhibitors, which are part of immunotherapeutic approaches. Emerging therapeutic strategies for the lysis of senescent cells are garnering significant interest within the oncology treatment field. Senomorphics and senolytics therapies are anticipated to emerge as the preferred treatment options for tumors in the future. Ultimately, considering the association between aging and tumorigenesis, there is anticipation for the future development of novel drugs that can enhance genomic stability or trigger programmed cell death in cancer cells. These treatments have the potential to alleviate the side effects associated with the extensive damage from radiotherapy and chemotherapy, as well as the propensity for relapse with targeted therapies. Furthermore, it augments the body's resistance to aging and tumorigenesis. The development of these drugs is expected to offer innovative strategies for cancer treatment, focused on diminishing the adverse effects of current therapies and enhancing the quality of life for patients.

## Data Availability

No datasets were generated or analysed during the current study.
